# Epidemiology, Risk Factors and Diagnosis of Small Bowel Adenocarcinoma

**DOI:** 10.3390/cancers14092268

**Published:** 2022-05-02

**Authors:** Thomas Aparicio, Atanas Pachev, Pierre Laurent-Puig, Magali Svrcek

**Affiliations:** 1 Gastroenterology and Digestive Oncology, Saint Louis Hospital, Assistance Publique Hôpitaux de Paris (APHP), Université de Paris, 75010 Paris, France; 2 Radiology Department, Saint Louis Hospital, Assistance Publique Hôpitaux de Paris (APHP), Université de Paris, 75010 Paris, France; atanas.pachev@aphp.fr; 3 Department of Biology, Georges Pompidou Hospital, Assistance Publique Hôpitaux de Paris (APHP), Université de Paris, 75010 Paris, France; pierre.laurent-puig@parisdescartes.fr; 4 Department of Pathology, Saint Antoine Hospital, Assistance Publique Hôpitaux de Paris (APHP), Sorbonne Université, 75012 Paris, France; magali.svrcek@aphp.fr

**Keywords:** small bowel adenocarcinoma, Crohn’s disease, Lynch syndrome, enteroscopy, videocapsule endoscopy

## Abstract

**Simple Summary:**

Small bowel adenocarcinoma is a rare tumor. Diagnosis is often obtained at an advanced stage and prognosis remains poor. The aim of this review is to report the recent epidemiological and risk factor data related to small bowel adenocarcinoma. New diagnostic tools are also described in this review.

**Abstract:**

Adenocarcinomas of the small intestine are rare tumors but their incidence is increasing. There is a slight male predominance. The median age at diagnosis is the 6th decade. The most frequent primary location is the duodenum. There is no clearly identified environmental risk factor, but adenocarcinomas of the small intestine are associated in almost 20% of cases with predisposing diseases (Crohn’s disease, Lynch syndrome, familial adenomatous polyposis, Peutz–Jeghers syndrome and celiac disease).

## 1. Introduction

Adenocarcinomas of the small bowel are rare tumors but their incidence is increasing. The most frequent primary location is the duodenum. These cancers are more frequently associated with predisposing diseases than colon cancers and their carcinogenesis remains poorly understood. The diagnosis is most often made after a complication (hemorrhage or occlusion).

## 2. Incidence and Risk Factors

### 2.1. Epidemiology

Although the small bowel represents 75% of the length of the digestive tract and 90% of its mucosal surface, cancers of the small bowel remain rare and represent around 3% of digestive cancers according to recent US cancer statistics [1]. The incidence of small bowel cancers has slightly risen in recent decades. It increased from 1.18 per 100,000 in 1973 to 2.27 per 100,000 in 2004 in the United States [2]. Likewise, in France, the incidence increased during the period 1976–2001 [3] but also during the period 1996–2015 [4]. Four main histologic types are present in the small bowel: adenocarcinomas, neuroendocrine tumors, stromal tumors and lymphomas. Small bowel adenocarcinomas (SBAs) account for about 40% of small bowel cancers, at the same level as neuroendocrine tumors [3,4,5]. Incidence of SBA has increased in the United States and Europe [2,3,5], particularly SBA of the duodenum [5]. In a population-based study in France, the age-standardized incidence rate of SBA was 0.69 per 100,000 in the 1996–2000 period and increased to 0.8 per 100,000 in the 2011–2015 period in men and it increased from 0.37 per 100,000 in the 1996–2000 period to 0.51 per 100,000 in the 2011–2015 period in women [4]. In the Netherlands, the age-standardized incidence of SBA increased from 0.5 per 100,000 in 1999 to 0.7 per 100,000 in 2013 [5]. According to EUROCARE data, the annual number of new cases of SBA estimated in Europe is 3600 [6]. The duodenum is the most frequently affected segment, accounting for 55–82% of cases, followed by the jejunum (11–25%) and ileum (7–17%) [7]. The increasing incidence is mainly due to duodenum adenocarcinoma. In a population-based study the Netherlands, a twofold increase in duodenal cases was observed from the 1999–2003 period to the 2009–2013 period [5]. SBA is most often diagnosed during the sixth decade and a slight male predominance is observed [5,8,9]. Thus, despite SBA not being a significant public health concern, its increasing incidence requires more attention and efforts to improve the treatment of this orphan disease.

### 2.2. Carcinogenesis

The large discrepancy in the incidence between SBA and colorectal adenocarcinoma suggests lower exposure to carcinogens. The molecular abnormalities demonstrated in SBA are common with those found in colonic adenocarcinomas but with different frequencies for some of them, which reflect a distinct carcinogenesis. The prevalence of molecular abnormality is presented in Table 1. Loss of expression of the adenomatous polyposis coli (APC) protein causes deregulation of β-catenin, which accumulates in the cytoplasm and then in the nucleus and acts as a transcription factor that stimulates the expression of genes involved in cell proliferation. Mutations in the *APC* gene are considered to be one of the major early events in colorectal carcinogenesis. The prevalence of *APC* mutations in SBA is low, from 13 to 27%, depending on the series [9,10,11,12], unlike colorectal cancers where this mutation is found in nearly 80% of cases. It seems more common in tumors of the duodenum [12]. A mutation in the *TP53* gene has been detected in 38% to 58% of tumors [9,10,11,12], less commonly in duodenal tumors and in the case of DNA repair abnormality (dMMR phenotype) [12]. Mutation of *TP53* is associated with dismal prognosis [13]. A *KRAS* mutation is found in 43% to 56% of cases [9,10,11,12]. Other *RAS* mutations are present in less than 5% of tumors [12]. Overexpression of the HER2 protein is observed more rarely, unlike in adenocarcinoma of the stomach [8,14]. However, alteration or amplification of the *ERBB2* gene has been reported in 7% to 14% of tumors [9,10,11,12]. A study reported an association of *ERBB2* mutation and duodenal location [12] but this was not confirmed by other studies [9,10]. One study reported an association with *ERBB2* mutation and dMMR tumors [12]. Moreover, *ERBB2* mutation was associated with a dismal prognosis in one study [15] but not in a larger study [10]. The *BRAF* mutation frequency ranges from 4% to 11% [9,10,11,12] but the majority of *BRAF* mutations were not the V600E, the most prevalent one in colorectal cancers. Mutation of *BRCA2* was reported in 5% of the tumors in one study [9]. Overall, a potentially targetable alteration was reported in 90% of SBAs in one study [11]. Nevertheless, a confirmation of the efficacy of targeted therapy remains to be demonstrated in SBA treatment. The presence or absence of a predisposing disease can modify the mutational landscape of SBA. Thus, *ERBB2* tumor mutations were more frequent in Lynch syndrome than in Crohn’s disease, and *TP53* mutations and *IDH1* mutations were more frequent in Crohn’s disease [10]. SMAD4 mutations were also associated with Crohn’s disease in one study [16]. A dMMR phenotype is found with a variable frequency, according to a study, in 5 to 35% of cases [7]. Methylation of the *MLH1* gene promoter appears to be less frequently involved in SBA than in colorectal cancers, suggesting that the dMMR phenotype is more frequently linked to Lynch syndrome [17]. The dMMR phenotype is more common in duodenal or jejunum tumors than in ileum tumors [8]. A dMMR phenotype was associated with a better prognosis [10,18]. Finally, an analysis of the exome of 106 SBAs found differences in mutational profiles between different segments [9]. Altogether, the discrepancy in molecular abnormality according to different small bowel segments in different studies is an issue. Larger studies with a pooled database are needed to establish the molecular phenotype of SBA according to localization and etiologic factors. The prognostic and theragnostic value of molecular abnormalities is a major issue to be assessed in the near future.

### 2.3. Risk Factors for Small Bowel Adenocarcinoma

The rarity of the pathology makes epidemiological studies difficult. Several hypotheses try to explain the low incidence of SBA. Owing to the shorter transit time in the small bowel compared to the colon, dietary carcinogens and xenobiotics have a shorter contact time with intestinal cells. Moreover, there is a lower concentration of aerophilic Gram-positive bacteria in the small bowel compared to the colon, even if the microbiota increase in the distal ileum [19]. Basal reactive oxygen species levels and antioxidant enzyme activities are lower in the small bowel than in the colon. Moreover, DNA adducts are more common due to oxidative stress in the colon than in the small intestine and therefore there is lower risk of small bowel transformation [20]. Finally, the epithelial cells of the small intestine have enzymatic equipment, in particular benzopyrene hydroxylases, which can protect against certain carcinogens [21]. The highest frequency of duodenal adenocarcinoma compared to distal SBA may be explained by a chronic irritation of acid gastric chyme as well as irritation by bile and pancreatic enzymes.

The presence of a predisposing disease or a genetic syndrome appears more frequently in SBA than in colorectal adenocarcinoma, and reached 20% in a large cohort [22]. Nevertheless, in the majority of cases no predisposing disease is involved.

#### 2.3.1. Lifestyle Factors

Evidence of lifestyle factors is difficult to obtain due to low incidence of the disease. A few studies give some conflicting results [23,24,25,26,27]. A systematic review of lifestyle risk factors and SBA suggests that alcohol intake (highest versus lowest category: 1.51 [95% CI: 0.83–2.75]) and smoking (highest versus lowest category: 1.24 [95% CI: 0.71-2.17]) are associated with a higher risk of SBA [28]. The systematic review also suggests that high fiber intakes and normal body weight may be protective, while high intakes of red/processed meat and sugary drinks may increase the risk of SBA [28]. Occupation risk factors were explored by one study that suggested that building caretakers, housekeepers, general farm laborers, dockers, dry cleaners, textile workers and welders were at risk for SBA [29]. Nevertheless, this explorative study needs further evaluation. Indeed, obtaining evidence of lifestyle factors or specific carcinogens for this rare disease is challenging.

#### 2.3.2. Genetic Syndromes

Lynch syndrome is a hereditary syndrome resulting from germline mutations in DNA mismatch repair genes (*MLH1*, *MSH2*, *MSH6* and *PMS2*). SBA is part of Lynch syndrome. However, the cumulative risk of developing this tumor remains low in patients with Lynch syndrome. It is estimated at 1% according to the ERISCAN study [30]. In a large recent study on Lynch syndrome, a gene-specific cumulative cancer risk for duodenal adenocarcinoma was reported, which was 6.5% for *MLH1* and 2.0% for *MSH2* carriers, but no SBA mutation was observed in patients with *MSH6* or *PMS2* mutations [31]. It is recommended to thoroughly explore the entire duodenum and distal ileum during the usual control endoscopies but not systematic exploration by capsule endoscopy [32]. However, an SBA can reveal Lynch syndrome [33] which implies that an MMR phenotyping must be systematically carried out for all SBAs according to guidelines [34,35]. In the NADEGE cohort, Lynch syndrome was reported in 7% of patients. The tumors were located in the duodenum in 61% of cases, the jejunum in 30% of cases and the ileum in 9% of cases [22] (Table 2). The localization of SBA in the jejunum should be kept in mind during the follow-up of a patient with Lynch syndrome and the whole small bowel should be explored in case of symptoms such as bleeding, anemia or unexplained abdominal pain.

Familial adenomatous polyposis (FAP) is related to mutation of the *APC* gene that results in numerous colon polyps and colorectal adenocarcinoma. Adenocarcinoma of the duodenum and adenocarcinoma of the ampulla of Vater are the second tumor localizations after colon cancer and they are the main cause of death [36]. A large registry study of patients with FAP reports that 4.5% of the patients develop an upper digestive tract adenocarcinoma. Among them, the most frequent primary location was duodenum in 50% of cases, follow by Vater ampulla in 18%, stomach in 12%, jejunum in 8.5% and ileum in 1.7% [37]. A tight screening for adenoma in the duodenum is recommended for individuals affected by FAP throughout their lifetime [38]. In another study, the relative risks for duodenal adenocarcinoma or ampulloma adenocarcinoma in FAP patients compared to the general population were 330 and 123, respectively [39]. FAP was reported in 2% of patients in the NADEGE cohort, 5/6 of patients had a duodenum tumor and 1/6 a jejunum tumor [22]. Despite the fact that SBA beyond the duodenum is rare in FAP, the exploration of all the small bowel is indicated in case of relevant symptoms and normal upper endoscopy.

Peutz–Jeghers syndrome is a rare autosomal dominant syndrome caused by a mutation in the tumor suppressor gene *STK11* which greatly increases the risk of developing SBA. The relative risk of developing SBA has been estimated as 520 compared with the general population [40]. The lifetime incidence for adenocarcinoma is 1.7–13% and rises rapidly in older patients [41]. However, this syndrome remains a rare etiology of SBA, and in the NADEGE cohort only two cases, i.e., 0.6% of patients, were reported [22]. Some cases of SBA have also been reported in juvenile polyposis syndrome related to *SMAD4* or *BMPR1A* mutation [41].

#### 2.3.3. Other Predisposing Diseases

Crohn’s disease is characterized by chronic inflammation of digestive tract mucosae. The colon and distal small bowel are the most frequently involved digestive tract segments. SBA arises most frequently in the distal ileum and in young patients in contrast to sporadic SBA [22]. A retrospective small series of SBA related to Crohn’s disease estimated the cumulative risk to be 0.2% after 10 years and 2.2% after 25 years of Crohn’s disease [42]. A large cohort study of 11,759 patients with Crohn’s disease estimated the standardized incidence ratio for SBA in patients with small bowel Crohn’s disease. It was 34.9 (95% CI, 11.3–81.5) in all patients and 46.0 (95% CI, 12.5–117.8) in patients suffering from Crohn’s disease for more than 8 years. This level of risk corresponds to one third of the risk of developing adenocarcinoma of the colon in Crohn’s disease affecting the colon [43]. Patients who have had resection of small intestine segments or who have prolonged treatment with salicylate have a lower risk of developing SBA [44]. Dysplasia is found near the adenocarcinoma in 49% of cases [45]. Crohn’s disease was reported in 9% of patients included in the NADEGE cohort. The median age of the patients is only 48 years and the tumor location is the duodenum in 7% of cases, the jejunum in 10% of cases and the ileum in 83% of cases [22]. The SBAs arising in Crohn’s disease have an aggressive phenotype with frequent metastases and a frequent poor differentiation [22] (Table 2). SBA should be suspected in patients with prolonged Crohn’s disease affecting the small bowel and that presents a worsening of symptoms or a resistance to usual treatment.

Celiac disease is associated with a relative risk of SBA compared to the general population which is estimated at 10 in a Swedish registry study [46]. A larger recent Swedish nationwide cohort of individuals with celiac disease reported a hazard ratio for SBA of only 3.05 [47]. In a large Italian cohort of celiac disease, 0.65% of the patients developed SBA [48]. In the NADEGE cohort, celiac disease was reported in 1.7% of the patients [22]. Celiac disease should be systematically screened after diagnosis of SBA as the cancer could reveal celiac disease with mild symptoms.

## 3. Clinical Presentation and Diagnostic Workup

### 3.1. Clinical Presentation

Due to the rarity of the disease, there is no screening program for SBA diagnosis. The symptoms are non-specific. In a study of 217 patients with SBA, most of the patients (66%) had abdominal pain at the time of diagnosis. Emergency diagnosis with occlusion or bleeding was reported in 40% and 24%, respectively. Symptoms differ according to localization. There is less bowel obstruction in case of duodenal tumors compared to jejuno-ileal tumors (34% vs. 47%; *p* = 0.06). At the time of this study (1978 to 1998), the diagnosis was made mainly by upper endoscopy (28%), during surgery (26%), by a small bowel barium transit (22%), by a CT scan (18%), ultrasound examination (3%) or physical examination (3%) [49]. In a more recent Japanese multicenter study, 43% of the duodenum adenocarcinomas were diagnosed without symptoms but this may be related to the gastric cancer screening ongoing in Japan [50]. In the NADEGE cohort, the contribution to diagnosis varied according to small bowel segment. Upper endoscopy gave a diagnosis for 49% of the duodenal adenocarcinoma, colonoscopy for 41% of the ileum adenocarcinoma and capsule endoscopy or CT scan with enteroclysis for 26% and 34% of the jejunum adenocarcinoma [22]. In Crohn’s disease, the diagnosis is frequently reached postoperatively after resection of an obstructed small bowel segment [42].

In the event of occult bleeding, exploration by capsule endoscopy has a sensitivity of 88.9% to 95% and a specificity of 95% to 75% for detecting a tumor of the small intestine [51,52]. Exploration by capsule endoscopy should not be performed in the event of a sub-occlusive syndrome and verification of the permeability of the small intestine with a patency capsule should be performed in the slightest doubt. For patients with intestinal polyposis syndromes, capsule endoscopy may be used to screen jejuno-ileal polyps [53]. A study that compared capsule endoscopy with magnetic resonance enterography reported that capsule endoscopy misses large polyps more often than magnetic resonance enterography [54]. Moreover, capsule endoscopy may miss tumors located in the duodenum or proximal jejunum because of rapid transit [55]. Improvement of imaging techniques may allow differential tumor type diagnosis, as was suggested by a small study assessing SBA and primary small bowel lymphoma with spectral CT imaging [56]. According to a prospective study on 150 patients with a suspected small bowel disease, magnetic resonance (MRI) enterography is more accurate than CT enterography for tumor detection [57]. Double balloon enteroscopy can then make it possible to obtain a preoperative histological diagnosis if necessary [58]. In patients with polyposis syndrome, device-assisted enteroscopy may be useful to remove polyps to prevent malignant transformation, bleeding or obstruction or to tattoo lesions before surgery [53]. Nevertheless, it must be pointed out that despite the new tools for diagnosis, there is no improvement in early diagnosis of SBA according to the result of a population-based study in the Netherlands [5].

Even in patients with predisposing disease, the early diagnosis is a challenge and no specific screening is recommended. In Crohn’s disease, the diagnosis of small bowel adenocarcinoma is often difficult because the symptoms are similar to those of the underlying pathology [42]. A chronic small bowel sub-obstruction that is not improved by medical treatment should be considered for surgical resection. In Lynch syndrome, two studies have evaluated videocapsule endoscopy for small bowel neoplasia screening. A prevalence of 8.6% of neoplasia (including adenomas and cancers) was reported in one study [59] but in another study the screening by capsule endoscopy had a low diagnostic yield with evidence of a small bowel tumors in only 1.5% [60]. In those studies, duodenal location was the most frequent. Thus, in Lynch syndrome, it is not recommended to perform systematic capsule endoscopy screening but systematic exploration of the duodenum should be performed when an upper endoscopy is indicated. In FAP, it is recommended to systematically screen only ampullary and duodenal polyps [39].

### 3.2. Initial Workup and Staging

Guidelines recommend an initial basic workup and some more specific examinations according to localization or predisposing disease [34,35]. The basic assessment includes a contrast-enhanced thoraco-abdomino-pelvic CT scan to evaluate local and metastatic extension. Liver MRI may be useful in case of contra-indication for iodine contrast agent or if liver metastases are suspected on CT scan examination. If possible, liver MRI should be performed before biliary drainage or stenting to minimize artifacts. Positron emission tomography (PET) scanning is not routinely indicated but may considered if there is doubt about metastasis on initial CT staging [61]. A gastric endoscopy and colonoscopy looking for other tumors are indicated in case of suspicion of genetic predisposition. In case of duodenal adenocarcinoma, an endoscopic ultrasound is recommended to assess vascular invasion and discern duodenal lesions from ampullary, biliary or pancreatic primary [62].

Dosage of CEA and CA 19-9 is useful at the initial workup, particularly in the event of a metastatic tumor, due to their prognostic value [34]. In Crohn’s disease, exploration of the entire intestine with MRI enterography or capsule endoscopy should be done to diagnose other synchronous tumor lesions. Testing for anti-transglutaminase A antibodies and duodenal biopsies are routinely recommended to detect celiac disease. Systematic screening for microsatellite instability or loss of expression of one of the MMR proteins should be systematically carried out to screen for Lynch syndrome and, for it, prognostic and predictive value for immunotherapy [10].

After diagnosis, follow-up with clinical examination, imaging and tumor marker dosage for a total duration of 5 years are recommended after a curative resection [34,35].

Staging is based on TNM staging (Table 3). It is recommended to assess a minimum of eight lymph nodes to have an adequate staging [34,35]. In western countries, the repartition of stages at diagnosis is: stage I (T1–T2, N0, M0) in 5% to 8%, stage II (T3–T4, N0, M0) in 20% to 29%, stage III (T1–4, N1–2, M0) in 24% to 29% and stage IV (M1) in 33% to 36% [5,22,63]. This is in contrast to a Japanese series that reported early stage diagnosis with 33% of the patients diagnosed at stage I, 17% at stage II, 24% at stage III and 26 at stage IV [50]. It must be pointed out that, in this study, early stage diagnosis was mainly reported for duodenal primary that could be explained by a screening program of gastric cancer in Japan. In the NADEGE cohort, there is no difference in stage at diagnosis according to predisposing disease or primary localization except 9% of unresectable localized tumors in duodenum primary with unknown precise staging [22].

### 3.3. Histological Diagnosis

Small intestine adenocarcinomas can occur anywhere in the gastrointestinal tract between the pylorus and the ileocecal valve [65]. In recent years, ampullary tumors have been considered a separate entity from head of pancreas and lower biliary tract tumors [66]. The region of ampulla is a crossroads between intestinal-type and pancreatobiliary-type epithelia. Thus, clinically and pathologically, it can be very challenging to distinguish between a tumor of the ampulla, an extra-ampullary duodenal tumor, or a metastatic tumor from the immediately neighboring regions of bile duct and pancreas. In this case, a careful macroscopic examination and dissection of pancreatoduodenectomy specimens is crucial and a tumor is considered to be of ampullary origin when its epicenter is the ampulla [67]. In the same way, the criterion for defining the duodenal origin of a tumor arising in the vicinity of the ampulla is the lack of both gross and microscopic involvement of the ampulla [68].

Histologically, small intestine adenocarcinomas resemble their colorectal counterparts. However poorly differentiated tumors with glandular, squamous and undifferentiated components are more frequently observed [65]. Extra-ampullary duodenal adenocarcinomas demonstrate histologic heterogeneity and can be divided into two major phenotypes: (i) intestinal-type adenocarcinomas, morphologically similar to their colorectal counterpart, and (ii) gastric-type adenocarcinomas, commonly associated with gastric foveolar metaplasia (63% of the cases) or Brunner gland hyperplasia (53% of the cases), which are two characteristics only observed in that setting The frequent association with adenomas for intestinal-type adenocarcinomas could suggest that duodenal adenocarcinomas develop in pre-existing adenomas, with an adenoma-carcinoma sequence similar to that described in colorectal carcinogenesis [69]. The intestinal type is associated with longer survival. Intestinal-type adenocarcinomas generally express CDX-2, MUC2 and CD10, whereas the gastric-type adenocarcinomas express MUC5AC and MUC6 [68]. Immunohistochemistry is not required to distinguish the gastric and intestinal phenotypes, but may be helpful in challenging cases.

It can also be difficult to distinguish between a metastatic adenocarcinoma, notably of colorectal origin, and a primary small intestine adenocarcinoma. In this case, the presence of a pre-cancerous lesion adjacent to the invasive lesion cannot be used to make a decision, because the small intestinal mucosa tends to mimic a pre-existing adenoma.

Tumors arising in the region of the ampulla may have either intestinal or pancreatobiliary type of differentiation. However, about 40% of cases harbor mixed or hybrid phenotypes. Panels of immunohistochemistry including MUC1, MUC2, CDX2, CK20 and MUC5AC can help in the classification of adenocarcinomas as intestinal or pancreatobiliary type in a substantial proportion of cases [70].

In the NADEGE cohort, around 80% of SBAs are well or moderately differentiated, but it must be pointed out that a poor differentiation is observed in 40% of Crohn’s disease cases, around 30% of tumors related to Lynch syndrome and 37% of ileum tumors [22].

## 4. Conclusions

In conclusion, SBA is a rare disease but with an increasing incidence. Duodenal adenocarcinoma may be diagnosed with upper endoscopy that should explore, as far as possible, the whole duodenum in case of macroscopic or occult bleeding. Except in FAP, no systematic screening is recommended in case of other predisposing diseases but clinicians should be aware of the risk of SBA. Early diagnosis remains infrequent and most SBAs are diagnosed at an advance stage. Several molecular abnormalities may be targeted by specific drugs. Currently, only immunotherapy in case of dMMR tumors has demonstrated significant efficacy. A global effort is needed to better understand risk factors and early carcinogenesis of this orphan disease.

## Figures and Tables

**Table 1:** Main molecular alterations observed in small bowel adenocarcinoma.

Publication	N	*KRAS* Mutation	*TP53* Mutation	PIK3CA Mutation	*APC* Mutation	*SMAD 4* Mutation	*ERBB2* Overexpression or Mutation	dMMR Phenotype
Aparicio et al. (2021)	125	44%	38%	20%	18%	14%	7%	28%
Hänninen et al. (2018) *	91	47%	48%	9%	24%	15%	14%	14%
Schrock et al. (2016)	358	56%	58%	16%	27%	17%	8%	7%
Laforest et al. (2014)	83	43%	41%	9%	13%	9%	12%	21%

* Gene mutation frequency is reported only in pMMR tumors.

**Table 2:** Differences according to predisposing disease observed in the NADEGE cohort [22].

Tumor Characteristics	No Predisposing Disease *n* = 278	Crohn’s Disease *n* = 30	Lynch Syndrome *n* = 24	*p*-Value
Median age (range)	64 (24–90)	48 (33–82)	56 (23–74)	<0.0001
Primary: Duodenum	66.7%	6.9%	60.9%	<0.0001
Jejunum	19.9%	10.3%	30.4%	
Ileum	13.4%	82.8%	8.7%	
Differentiation: Well and moderate	81.6%	60.7%	68.2%	0.0166
Poorly differentiated	18.4%	39.3%	31.8%	

**Table 3:** TNM staging from 8th edition 2017 [64].

**Primary tumor (T)** T0 There is no evidence of a primary tumor Tis Carcinoma in situ T1 Tumor invades the mucosa, muscularis mucosa or submucosa T1a Tumor invades the lamina propria or muscularis mucosa T1b Tumor invades the submucosa T2 Tumor invades the muscularis propria T3 Tumor invades the subserosa or into the non-peritonealized perimuscular tissue (mesentery or retroperitoneum) T4 Tumor perforates the visceral peritoneum (T4a) or directly invades other organs or structures (T4b), including: - other loops of the small intestine, mesentery or retroperitoneum - through the serosa into the abdominal wall - the pancreas (only for tumors in the duodenum)
**Regional lymph nodes (N)** N0 No regional lymph node metastasis N1 Metastasis in 1–2 regional lymph nodes N2 Metastasis in 3 or more regional lymph nodes
**Distant metastasis (M)** M0 No distant metastasis M1 Distant metastasis
**Cancer staging** Stage 0: Tis, N0, M0 Stage I: T1 or T2, N0, M0 Stage IIA: T3, N0, M0 Stage IIB: T4, N0, M0 Stage IIIA: any T, N1, M0 Stage IIIB: any T, N2, M0 Stage IV: any T, any N, M1
